# The Value of Routine Histopathology After Laparoscopic Cholecystectomy: A Single-Centre Retrospective Analysis

**DOI:** 10.7759/cureus.95453

**Published:** 2025-10-26

**Authors:** Abdelrahman Elafandy, Ayatullah Motawe, Amjad Riaz

**Affiliations:** 1 General Surgery, Warwick Hospital, Warwick, GBR; 2 Ophthalmology, Shebin Elkom Ophthalmology Hospital, Shebin Elkom, EGY; 3 Surgery, Warwick Hospital, Warwick, GBR

**Keywords:** cancer gallbladder, gall bladder histopathology, lap cholecystectomy, routine histopathology, selective system

## Abstract

Introduction

Laparoscopic cholecystectomy is among the most common general surgical procedures globally. While many centers routinely send all gallbladder specimens for histopathological examination, the clinical and cost-effectiveness of this practice is debated. This study evaluates the utility of a routine approach compared to a selective one.

Methods

We conducted a retrospective observational study of 171 patients (n=171) who underwent elective, expedited, or urgent laparoscopic cholecystectomy at Warwick Hospital between 1 July 2023 and 30 January 2024. Data were extracted from the theatre management system (ORMIS) and electronic patient records (E-volve).

Results

The cohort comprised 118 females (69%) and 53 males (31%), with a mean age of 53.1 years (±14.6). Most procedures were elective (73.7%, n=126), followed by expedited (14.6%, n=25) and urgent (11.7%, n=20). Histopathological analysis identified a single case (0.58%, n=1) of low-grade dysplasia with clear margins, which required no further intervention. The vast majority of specimens (99.4%, n=170) confirmed benign inflammatory disease.

Conclusion

The incidence of unexpected, clinically significant histopathological findings is exceptionally low. Subjecting all cholecystectomy specimens to routine histopathology imposes a substantial financial burden on the healthcare system with minimal patient benefit. A selective policy, guided by intraoperative macroscopic appearance and patient risk factors, represents a more rational and cost-effective strategy.

## Introduction

Laparoscopic cholecystectomy stands as one of the most frequently performed procedures in general surgery, serving as the definitive treatment for symptomatic cholelithiasis and its complications [1]. Its high volume makes it a significant contributor to both surgical workload and overall healthcare expenditure. The procedure is indicated across a spectrum of clinical presentations, ranging from chronic biliary colic to complicated cholecystitis.

In many institutions, including the National Health Service (NHS), it is standard practice to submit all excised gallbladders for routine histopathological analysis. This protocol aims primarily to detect occult pathology, with incidental gallbladder carcinoma (IGBC) being the most critical, though rare, finding in Western populations, ranging between 0.14% and 2% [1,2]. The impetus for this universal policy often stems from a combination of clinical vigilance, defensive medicine, and established tradition.

Clarifying the terminology of surgical urgency is essential. 'Elective' procedures are planned operations for chronic symptoms. 'Expedited' or 'Hot' cases are performed during the index admission for an acute presentation, while 'Urgent' typically refers to emergency surgery for severe complications, such as empyema or gangrenous cholecystitis. The perceived risk of underlying malignancy may be higher in non-elective cases.

The current standard of care in our trust, and many others, mandates histopathological examination of every gallbladder specimen. This universal approach incurs considerable direct and indirect costs, including laboratory processing, pathologist time, and administrative resources [2]. In an era of intense scrutiny on healthcare efficiency and cost-containment, it is imperative to critically evaluate practices that consume considerable resources for a potentially negligible clinical yield.

This study addresses whether the routine 'send all' policy represents high-value care. The financial burden of processing hundreds of specimens annually to identify a minute number of actionable results is a pressing concern. These resources could be reallocated to reduce patient waiting times or fund other services with a more demonstrable impact on outcomes [3].

Therefore, we conducted a rigorous, data-driven evaluation of the value of routine histopathology following laparoscopic cholecystectomy. By analyzing the incidence and clinical impact of significant incidental findings, we aim to provide evidence to inform a potential shift towards a more selective and cost-effective policy without compromising patient safety.

## Materials and methods

Study design and population 

This single-centre retrospective observational study included all consecutive patients who underwent laparoscopic cholecystectomy at Warwick Hospital, Warwick, England, between 1 July 2023 and 30 January 2024, irrespective of urgency classification (elective, expedited, or urgent).

Inclusion criteria: We included patients of all ages and genders who underwent laparoscopic cholecystectomy for symptomatic gallstones. All the National Confidential Enquiry into Patient Outcome and Death (NCEPOD) classifications were included.

Exclusion criteria: Patients who underwent laparoscopic cholecystectomy for a pre-operatively diagnosed gallbladder malignancy were excluded.

A comprehensive list of eligible patients was generated from the hospital's theatre management system (ORMIS). This list was cross-referenced with the institution's electronic patient record system (E-volve) to ensure completeness. The following data points were meticulously extracted for each patient: demographic information (age, sex), procedure date, surgical urgency (as defined by NCEPOD criteria), and the final histopathology report.

Data were anonymized and collated. Descriptive statistics were calculated for demographic and clinical variables, presented as means with standard deviations (SD) for continuous data and counts with percentages for categorical data. The primary outcome was the proportion of specimens with clinically significant histopathology (defined as malignancy, dysplasia, or any finding that altered patient management). A chi-squared goodness-of-fit test was used to determine if the observed rate of significant pathology differed from a hypothesized population rate of 5%. Statistical analysis was performed using Microsoft Excel (Microsoft® Corp., Redmond, WA), with a significance level set at p < 0.05.

## Results

Over the seven-month study period, 171 patients (n=171) underwent laparoscopic cholecystectomy. The demographic and clinical characteristics of the cohort are summarized in Table 1.

**Table 1:** Patient demographics and case distribution (n=171)

Characteristic	Value
Gender, n (%)	
Female	118 (69.0%)
Male	53 (31.0%)
Female-to-Male Ratio	2.2:1
Age (Years)	
Mean ± Standard Deviation	53.1 ± 14.6
Theatre Duration (Minutes)	
Mean ± Standard Deviation	80.4 ± 37.5
Case Distribution by Urgency	
Elective	126 (73.7%)
Expedited	25 (14.6%)
Urgent	20 (11.7%)

**Figure 1:**
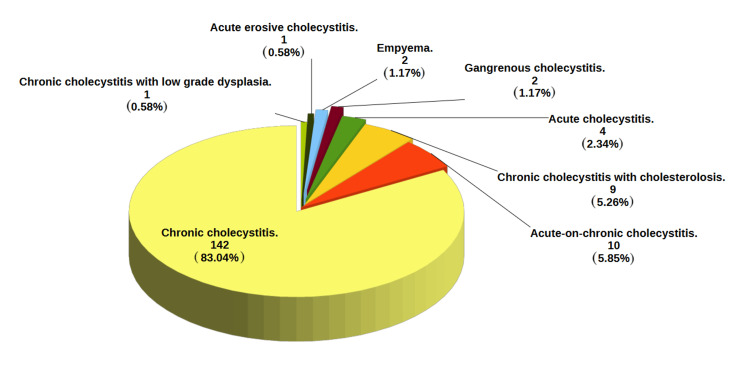
Histopathological findings in 171 gallbladder specimens

The distribution of cases by urgency is demonstrated in Table 1. The detailed histopathological findings are presented in Figure 1. Most specimens (170, 99.4%) confirmed a spectrum of benign inflammatory disease, with chronic cholecystitis being the predominant diagnosis (142, 83%).

The single case of low-grade dysplasia was reviewed by the gastroenterology multidisciplinary team (MDT), and no further intervention was required.

A chi-squared goodness-of-fit test was performed, comparing the observed rate of significant pathology (1/171, 0.6%) against a hypothesized rate of 4.6% in 100,000 of the population [4]. The result was statistically significant (χ² = 13.41; p < 0.001), confirming that the actual yield of actionable pathology is significantly lower.

Based on the volume of cases in this study, the estimated annual cost to Warwick Hospital for routine histopathology for laparoscopic cholecystectomy specimens is about £37,500.

## Discussion

This study provides a compelling argument against the routine histopathological examination of gallbladder specimens following laparoscopic cholecystectomy. Our data indicate that, in 99.4% of cases, the histology report merely confirms a benign inflammatory condition that was anticipated from clinical and intraoperative findings. This reduces the practice to a costly formality for the overwhelming majority of patients and the healthcare system. The identification of a single case of low-grade dysplasia (0.6%), which did not alter clinical management, starkly illustrates the exceptionally low yield of this universal policy.

The financial implications are substantial. An annual expenditure of £37,500 represents a significant investment in a single, low-yield diagnostic test. When extrapolated across the NHS, this equates to millions of pounds annually spent on confirming predictable diagnoses. These resources are effectively diverted from other areas of patient care where they could have a greater impact, such as reducing waiting times for cancer diagnostics or elective surgery. This practice, therefore, raises serious questions about opportunity cost within a financially constrained system [1].

Critics of a selective policy may argue that routine histology provides a definitive diagnosis and mitigates litigation risk. However, a health-economic analysis reveals this practice to be a poor investment. The annual cost of £37,500 for routine histology in our trust must be weighed against its minimal clinical yield. The incidence of incidental gallbladder cancer (IGBC) requiring consequential action is exceedingly low, estimated at just 0.5-1.0% [1] up to 2% [2]. In our cohort, this translated to a single non-actionable finding (0.58%). Furthermore, a selective policy avoids the significant downstream costs of false positives; transabdominal ultrasound has a false-positive rate for true polyps of up to 85%, often leading to unnecessary follow-up imaging and patient anxiety [5]. When these factors are modelled, the cost-per-clinically-significant-finding of a routine policy becomes prohibitive. From a medico-legal perspective, a protocol-driven selective approach based on established risk factors - such as macroscopic suspicion [5,6], specific patient demographics [7], or the presence of primary sclerosing cholangitis [3] - is defensible. This strategy aligns with modern management principles, where intraoperative frozen section for suspicious cases can guide single-stage definitive surgery, avoiding the poorer outcomes associated with unplanned conversion or re-operation [6,8]. Therefore, a selective policy is not only cost-effective but also represents a more sophisticated, evidence-based standard of care that focuses resources on high-risk scenarios, ultimately enhancing patient safety.

The management of the single dysplastic lesion in our cohort further supports a selective approach. Even when identified, the MDT determined that no further action was required. This aligns with the literature, which indicates that, for dysplastic lesions and T1a carcinomas with clear margins, the cholecystectomy itself is curative [6]. Therefore, the clinical consequence of identifying such a lesion in an otherwise normal-appearing gallbladder is often negligible, further undermining the argument for routine examination.

Advancements in minimally invasive surgery have further strengthened the case for selective histology. A comparative study by Lee et al. demonstrated that laparoscopic extended cholecystectomy for gallbladder cancer is oncologically safe for early-stage disease, with no port-site recurrences and comparable long-term survival to open surgery [9]. This finding is critical as it mitigates historical concerns that laparoscopic manipulation might disseminate tumor cells, a fear that previously fueled demands for routine histology. The demonstrated safety of laparoscopy for known cancer reinforces that a surgeon's macroscopic assessment during a routine procedure is a reliable tool, thereby validating a selective policy based on gross appearance.

The clinical drawbacks of failing to identify high-risk cases pre-emptively are highlighted by research into converted procedures. Silva et al. found that patients with IGBC who required conversion from laparoscopic to open cholecystectomy had significantly worse postoperative outcomes, including longer hospital stays and higher rates of sepsis and reoperation [8]. This underscores a key benefit of a selective system: the intraoperative identification of suspicious features allows the surgeon to adapt the procedure at the index operation, potentially avoiding a second intervention or a complicated conversion. A policy that encourages meticulous macroscopic examination empowers the surgeon to make this critical decision intraoperatively, thereby streamlining care and averting the heightened morbidity associated with unplanned conversions.

The cornerstone of an effective selective policy is the ability to accurately identify suspicious features intraoperatively, a skill supported by radiological-pathological correlation. The comprehensive review by Riddell et al. delineates specific imaging characteristics that stratify malignancy risk in gallbladder polyps and wall thickening [3]. Features such as size ≥10 mm, sessile morphology, solitary polyps, and demographic factors like primary sclerosing cholangitis or older age significantly increased the risk. This evidence provides a clear framework, translating radiological risk factors into tangible intraoperative criteria. Training surgeons to recognize the macroscopic correlates of these features moves the selective system from an abstract concept to a practical, evidence-based protocol.

Ultimately, the most compelling evidence for a selective policy comes from direct comparisons with routine practice. A systematic review by Khan et al., which analyzed over 77,000 cholecystectomies, found that in studies advocating for selective histology, the vast majority of incidental gallbladder carcinomas had been suspected by the surgeon during macroscopic examination [5]. Crucially, the review noted that many macroscopic findings missed by surgeons in 'routine' studies were successfully identified as suspicious in 'selective' studies. This indicates that the efficacy of a selective policy is intrinsically linked to the diligence and training of the operating surgeon. The authors concluded that 'selective histological examination of cholecystectomy specimens can be preferred if a careful intraoperative macroscopic examination is done and patient risk factors are taken into consideration' [9]. This directly validates the central thesis of our study and confirms that a well-implemented selective system is not a compromise, but a superior standard of care that combines clinical acumen with resource stewardship.

Our statistical analysis confirms that the incidence of significant pathology is not merely low but significantly lower than the historical benchmarks used to justify the 'send all' protocol. Modern surgical cohorts, particularly in Western populations, demonstrate a very low prevalence of IGBC, making the continued use of a universal policy increasingly difficult to defend on an evidence-based platform.

The limitation of this study is possibly the size of the population, as the incidence rate of gallbladder cancer is very low. A future randomized study with a larger population would overcome this limitation.

In conclusion, the data presented here make an incontrovertible case for change. The routine histopathological examination of gallbladder specimens is a high-cost, low-benefit practice that consumes valuable healthcare resources without delivering meaningful clinical value for the majority of patients. Implementing a selective policy is not merely a cost-saving measure; it is a fundamental step towards practicing rational, evidence-based, and high-value surgery.

## Conclusions

This analysis demonstrates that routine histopathological assessment of gallbladder specimens after laparoscopic cholecystectomy almost exclusively confirms anticipated benign disease (99.4% of cases), at an annual cost of approximately £37,500 to our trust. The single clinically relevant finding (0.6%) did not alter patient management.

Therefore, the continued practice of universal submission is financially unsustainable and clinically unjustifiable. The adoption of a selective policy, guided by specific intraoperative and patient-related criteria, is a safe, responsible, and economically imperative strategy that aligns with the principles of value-based healthcare.
